# Adherence to a stress ulcer prophylaxis protocol by critically ill patients: a prospective cohort study

**DOI:** 10.5935/0103-507X.20200007

**Published:** 2020

**Authors:** Yuri de Albuquerque Pessoa dos Santos, Mauricio Staib Younes-Ibrahim, Lucas Lonardoni Crozatti, Dante Raglione, Luis Carlos Maia Cardozo Junior, Bruno Adler Maccagnan Pinheiro Besen, Leandro Utino Taniguchi, Marcelo Park, Pedro Vitale Mendes

**Affiliations:** 1 Unidade de Terapia Intensiva, Departamento de Emergências, Hospital das Clínicas, Faculdade de Medicina, Universidade de São Paulo - São Paulo (SP), Brazil.; 2 Instituto de Ensino e Pesquisa, Hospital Sírio-Libanês - São Paulo (SP), Brazil.; 3 Unidade de Terapia Intensiva Oncológica, Hospital São Luiz, Rede D’Or - São Paulo (SP), Brazil.

**Keywords:** Therapeutic adherence compliance, Anti-ulcer agents, Peptic ulcer, Gastrointestinal hemorrhage, Critical care, Critical illness, Cooperação e adesão ao tratamento, Antiulcerosos, Úlcera péptica, Hemorragia gastrointestinal, Cuidados críticos, Estado terminal

## Abstract

**Objective:**

To evaluate adherence to the stress ulcer prophylaxis protocol in critically ill patients at a tertiary university hospital.

**Methods:**

In this prospective cohort study, we included all adult patients admitted to the medical and surgical intensive care units of an academic tertiary hospital. Our sole exclusion criterion was upper gastrointestinal bleeding at intensive care unit admission. We collected baseline variables and stress ulcer prophylaxis indications according to the institutional protocol and use of prophylaxis. Our primary outcome was adherence to the stress ulcer prophylaxis protocol. Secondary outcomes were appropriate use of stress ulcer prophylaxis, upper gastrointestinal bleeding incidence and factors associated with appropriate use of stress ulcer prophylaxis.

**Results:**

Two hundred thirty-four patients were enrolled from July 2nd through July 31st, 2018. Patients were 52 ± 20 years old, 125 (53%) were surgical patients, and the mean SAPS 3 was 52 ± 20. In the longitudinal follow-up, 1499 patient-days were studied; 1069 patient-days had stress ulcer prophylaxis indications, and 777 patient-days contained prophylaxis use (73% stress ulcer prophylaxis protocol adherence). Of the 430 patient-days without stress ulcer prophylaxis indications, 242 involved prophylaxis (56% inappropriate stress ulcer prophylaxis use). The overall appropriate use of stress ulcer prophylaxis was 64%. Factors associated with proper stress ulcer prophylaxis prescription were mechanical ventilation OR 2.13 (95%CI 1.64 - 2.75) and coagulopathy OR 2.77 (95%CI 1.66 - 4.60). The upper gastrointestinal bleeding incidence was 12.8%.

**Conclusion:**

Adherence to the stress ulcer prophylaxis protocol was low and inappropriate use of stress ulcer prophylaxis was frequent in this cohort of critically ill patients.

## Abstract (pt)

**Objetivo:**

Avaliar a adesão ao protocolo de profilaxia de úlcera de estresse em pacientes críticos de um hospital universitário terciário.

**Métodos:**

Neste estudo de coorte prospectiva, incluímos todos os pacientes adultos admitidos às unidades de terapia intensiva clínica e cirúrgica de um hospital terciário acadêmico. Nosso único critério de exclusão foi a presença de sangramento gastrointestinal alto quando da admissão à unidade de terapia intensiva. Colhemos as variáveis basais e indicações de profilaxia de úlcera de estresse, segundo o protocolo institucional, assim como o uso de profilaxia. Nosso desfecho primário foi a adesão ao protocolo de profilaxia de úlcera de estresse. Os desfechos secundários foram uso apropriado da profilaxia de úlcera de estresse, incidência de sangramento gastrointestinal superior e fatores associados com o uso apropriado da profilaxia de úlcera de estresse.

**Resultados:**

Foram incluídos 234 pacientes no período compreendido entre 2 de julho e 31 de julho de 2018. Os pacientes tinham idade de 52 ± 20 anos, sendo 125 (53%) deles cirúrgicos, e o SAPS 3 médio foi de 52 ± 20. No seguimento longitudinal, foram estudados 1.499 pacientes-dias; 1.069 pacientes-dias tiveram indicação de profilaxia de úlcera de estresse, e 777 pacientes-dias tiveram uso profilático (73% de adesão ao protocolo de profilaxia de úlcera de estresse). Dentre os 430 pacientes-dias sem indicações de profilaxia de úlcera de estresse, 242 envolveram profilaxia (56% de uso impróprio de profilaxia de úlcera de estresse). O total de uso apropriado de profilaxia de úlcera de estresse foi de 64%. Fatores associados com prescrição adequada de profilaxia de úlcera de estresse foram ventilação mecânica, com RC 2,13 (IC95% 1,64 - 2,75), e coagulopatia, com RC 2,77 (IC95% 1,66 - 4,60). A incidência de sangramento do trato gastrointestinal superior foi de 12,8%.

**Conclusão:**

A adesão ao protocolo de profilaxia de úlcera de estresse foi baixa, e o uso inadequado de profilaxia de úlcera de estresse foi frequente nesta coorte de pacientes críticos.

## INTRODUCTION

Critically ill patients are at risk for upper gastrointestinal bleeding (UGB) due to stress ulcer.^(1-3)^ The pathophysiology is not entirely understood: it has been hypothesized that splanchnic hypoperfusion, impaired microcirculation, and the proinflammatory state predispose patients to the disruption of the gastric mucosal barrier and the occurrence of stress ulcer.^(4,5)^ Clinically significant UGB in intensive care unit (ICU) patients is associated with severe adverse outcomes, including increased risk of death and increased ICU length of stay.^(1,2)^

Stress ulcer prophylaxis (SUP) was introduced more than 40 years ago to prevent UGB.^(6)^ Guidelines recommend acid suppressants for patients at high risk for UGB.^(7-9)^ Nevertheless, concerns regarding potential harms of acid suppression in the gastrointestinal microbiome^(10)^ are increasing, given its association with infectious complications such as nosocomial pneumonia^(11-13)^ and *Clostridioides difficile* infection.^(14-18)^ Furthermore, the use of SUP may be associated with drug-induced thrombocytopenia,^(19)^ myocardial infarction,^(20,21)^ hypomagnesemia^(22)^ and the risk of drug interaction.

Current meta-analyses - including studies of low quality of evidence - have shown that SUP reduces the incidence of overt bleeding with no effects on mortality,^(23,24)^ raising some doubts about its cost-effectiveness. Recently, a randomized, multicenter clinical trial with almost 3,300 critically ill patients demonstrated that pantoprazole lowered the rate of UGB without reducing mortality in comparison with placebo.^(25)^ Therefore, considering that SUP use may reduce gastrointestinal bleeding in critically ill patients but is possibly associated with significant adverse effects and increased costs, knowledge of proper adherence to SUP recommendations is fundamental for proper high-value care. In accordance with previous publications,^(26,27)^ we hypothesize that SUP prescription will be inadequate in this cohort of critically ill patients.

We conducted this study to evaluate the adherence to SUP in critically ill patients. As a secondary outcome, we evaluated UGB incidence and factors associated with proper use of SUP in this population.

## METHODS

This was a single-center, prospective cohort study in eight medical and surgical ICUs of *Hospital das Clínicas* of the *Faculdade de Medicina* of the *Universidade de São Paulo* (USP). It aimed to evaluate SUP adherence in critically ill patients. This teaching hospital is one of the largest hospital complexes in Latin America, with a total of 2,400 active beds, and acts as a referral center in the city of São Paulo. The study protocol was approved by the Research Ethics Committee of *Hospital das Clínicas* of the *Faculdade de Medicina* of the USP (number - 2.822.929). Because of the observational nature of the study, a waiver of informed consent was obtained.

All patients 18 years of age or older admitted to any of the eight intensive care units of *Hospital das Clínicas* of the *Faculdade de Medicina* of the USP between July 2nd and July 31st, 2018 were eligible for inclusion. Patients admitted with gastrointestinal bleeding were excluded.

The primary outcome was adherence to the SUP protocol. Secondary outcomes included the incidence of UGB and evaluation of factors associated with appropriate use of SUP.

Baseline data such as sex, age, Charlson comorbidity index, initial diagnosis, and Simplified Acute Physiology Score 3 (SAPS 3)^(28)^ score were collected at admission. During the ICU stay, SUP indications, SUP use, overt UGB occurrence, and UGB risk factor presence were collected daily. The SUP medications recommended by our institutional protocol were omeprazole and ranitidine. Both could be administered intravenously or by enteral formulation.

Overt UGB was defined as the presence of melena, hematemesis or endoscopic evidence of active gastrointestinal bleeding. However, an endoscopic evaluation was not routinely performed, nor was it mandatory for this diagnosis.

Upper gastrointestinal bleeding risk factors, following the institutional protocol and in accordance with a recent randomized clinical trial, were:^(25)^ shock (if vasopressors or inotropes were necessary); mechanical ventilation expected to last > 24 hours; renal-replacement therapy; 4) use of anticoagulant agents (prophylactic doses excluded); 5) chronic liver disease (cirrhosis, portal hypertension); and 6) ongoing coagulopathy (International Normalized Ratio - INR > 1.5, platelets < 50,000).

The density of SUP use opportunity was calculated as the sum of the number of days with at least one risk factor present among all patients enrolled in the study, and the metric unit presented was the patient-day.

The density of appropriate SUP use was calculated as the sum of the number of days of SUP use among patients with at least one UGB risk factor. The density of inappropriate SUP use was the sum of the number of days on SUP use among patients without UGB risk factors.

The overall SUP use was considered the sum of proper SUP use (appropriate and inappropriate) by all patients. The SUP use adherence was calculated as the ratio between the density of appropriate SUP use and the density of opportunity of SUP use.

### Statistical analysis

Descriptive statistics are presented as number (percentage), median (P25 - P75) or mean (± standard deviation).

For secondary analyses, we evaluated which risk factors were associated with proper SUP prescription through multiple binary logistic regression. Although all risk factors are, a priori, indicative of SUP prescription, it was possible that an individual factor was considered more relevant to the occurrence of UGB by the attending physician than others.

Variables were included in the model on the basis of clinical significance. The results are presented as point estimates with adjusted 95% confidence intervals. There was no imputation for missing data. We used STATA version 15.1 for all statistical analyses.

## RESULTS

**Figure 1:**
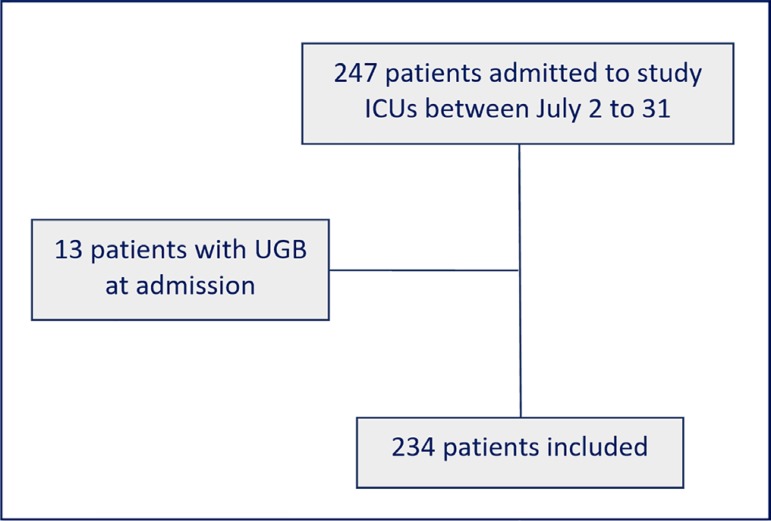
Study flowchart. ICU- intensive care unit; UGB - upper gastrointestinal bleeding.

Two hundred thirty-four patients were enrolled in the study from July 2nd to July 31st, 2018 (Figure 1). Upper gastrointestinal bleeding occurred in 30 patients (12.8%). The demographic characteristics of the patients at baseline were similar between the UGB and the non-UGB patients (Table 1).

**Table 1:** Patient characteristics

Characteristics	All patients (n = 234)	UGB (n = 30)	Non UGB (n = 204)	p value
Age (years)	52 (± 20)	51 (± 19)	52 (± 19)	0.72
Male	123 (52)	14 (52)	109 (53)	0.41
Charlson median	1 (0 - 3.0)	1 (0 - 3.5)	1 (0 - 2.0)	0.63
SAPS 3	52 (± 20)	58 (± 19)	52 (± 20)	0.42
Surgical admission	125 (53)	18 (67)	76 (36)	0.21
Mechanical ventilation	94 (40)	18 (67)	76 (36)	0.07
Vasoactive drug	96 (41)	14 (52)	82 (40)	0.29
Enteral nutrition	99 (42)	11 (41)	88 (42)	0.86
ICU LOS	7 (4.0 - 16.0)	13 (9.0 - 19.0)	6 (3.0 - 16.0)	0.04
ICU mortality	64 (27)	11 (36)	53(26)	0.95
Hospital LOS	16 (9.0 - 32.0)	16 (12.0 - 38.0)	16 (8.0 - 32.0)	0.89
Hospital mortality	78 (33)	13 (43)	65 (32)	0.81

UGB - upper gastrointestinal bleeding; SAPS 3 - Simplified Acute Physiology Score 3; LOS - length of stay; ICU - intensive care unit. The p-value represents comparison across both groups for each variable. Results expressed as mean (± standard deviation), n (%) or n (interquartile range).

The density of SUP use opportunity was 1499 patient-days. In 1069 of them, at least one UGB risk factor was present, but only in 777 patient-days was SUP prescribed (73% adherence). Of the 430 patient-days without at least one UGB risk factor, 242 patient-days included prophylaxis use (56% inappropriate use). The overall appropriate use was 64%, considering that 965 patient-days had proper SUP use.

Stress ulcer prophylaxis indications associated with adherence to SUP were mechanical ventilation (odds ratio - OR = 2.13; 95% confidence interval - 95%CI 1.64 - 2.75) and coagulopathy (OR = 2.77; 95%CI 1.66 - 4.60). Conversely, anticoagulant use was negatively associated with SUP prescription (OR = 0.47; 95%CI 0.29 - 0.84) (Table 2). Overt UGB occurred in 30 patients, corresponding to an incidence of 12.8%.

**Table 2:** Factors associated with adequate use of stress ulcer prophylaxis

SUP indications	Odds ratio (95%CI)	p value
Shock	0.89 (0.67 - 1.17)	0.42
Anticoagulants	0.49 (0.29 - 0.84)	0.009
Renal replacement	1.12 (0.84 - 1.50)	0.41
Mechanical ventilation	2.13 (1.64 - 2.75)	0.001
Coagulopathy	2.77 (1.66 - 4.60)	0.001
Liver disease	0.47 (0.15 - 1.43)	0.18

SUP - stress ulcer prophylaxis; 95%CI - 95% confidence interval.

## DISCUSSION

The adherence to the SUP protocol was low in this cohort of critically ill patients, and for every 3 ICU patients, one did not receive proper prophylaxis. Perhaps this might have occurred because SUP indications usually differ between society guidelines, and clinical trials did not contemplate some of the conditions associated with stress ulcer (i.e., traumatic brain injury; burns).^(29,30)^

Moreover, more than half of the patients without SUP indications were using prophylaxis. This is a significant concern since a significant proportion of patients who start using prophylaxis in the ICU continue its use inappropriately on the ward and even after hospital discharge.^(31,32)^ In addition, inappropriate use of SUP may be associated with higher costs, potential adverse events, and possible undesirable drug interactions.^(26,27,33)^ Improving prescribing awareness through greater involvement of clinical pharmacists, interdisciplinary education and compliance with institutional protocols have previously been shown to be effective and could be consistent approaches to reduce inappropriate SUP use.^(34,35)^

Our trial had similar results to previous studies,^(36,37)^ which also reported a high rate of inappropriate SUP prescription. In a cohort of patients admitted to the general ward of a teaching hospital, only 28.8% of SUP prescriptions were in accordance with local policy, and a high number of patients continued with SUP use even after discharge.^(37)^

It is interesting to note that the right time for discontinuing prophylactic use in patients who no longer carry a risk factor for stress ulcer in the ICU is unknown. Some reports have continued the use of SUP until ICU discharge,^(25,32)^ while other studies have ceased its use immediately after the risk factor went away.^(38,39)^ In our cohort, we chose to evaluate the presence of risk factors and consequent indications for SUP daily. This may explain the low adherence to the SUP protocol since the institutional protocol does not define the moment of SUP discontinuation.

Finally, invasive mechanical ventilation and coagulopathy were associated with a higher adherence for SUP use in our cohort. Conversely, anticoagulant use was negatively associated with SUP prescription. This may have occurred because earlier studies report only the association of invasive mechanical and coagulopathy with stress ulcer occurrence, without anticoagulation as a risk factor^(3)^ and because those are widely known risk factors in clinical practice.

Our study has several limitations. First, it was an observational, single-center study, and external validity is a major concern; however, it is representative of 8 different ICUs with different ICU practices and intensivists from different backgrounds. Second, we did not evaluate any adverse events related to prophylaxis use. Finally, the absence of endoscopic evaluation may have led us to overestimate the incidence of stress ulcer in our cohort. However, in a recent randomized controlled trial, the incidence of any overt UGB was 9% in the control group, similar to the rate in this cohort of critically ill patients.^(25)^

## CONCLUSION

The adherence to the stress ulcer prophylaxis protocol was low, and inappropriate use of stress ulcer prophylaxis was common in this cohort of critically ill patients.
